# Chronic Circadian Disruption and Sleep Restriction Influence Subjective Hunger, Appetite, and Food Preference

**DOI:** 10.3390/nu14091800

**Published:** 2022-04-26

**Authors:** Andrew W. McHill, Joseph T. Hull, Elizabeth B. Klerman

**Affiliations:** 1 Sleep, Chronobiology, and Health Laboratory, School of Nursing, Oregon Health & Science University, 3455 SW US Veterans Hospital Rd., Portland, OR 97239, USA; 2 Oregon Institute of Occupational Health Sciences, Oregon Health & Science University, Portland, OR 97239, USA; 3 Division of Sleep Medicine, Harvard Medical School, Boston, MA 02115, USA; jthboston@gmail.com (J.T.H.); elizabeth_klerman@hms.harvard.edu (E.B.K.); 4 Division of Sleep and Circadian Disorders, Departments of Medicine and Neurology, Brigham and Women’s Hospital, Boston, MA 02115, USA; 5 Department of Neurology, Massachusetts General Hospital, Boston, MA 02114, USA

**Keywords:** circadian misalignment, insufficient sleep, shiftwork, leptin, ghrelin

## Abstract

Chronic circadian disruption (CCD), such as occurs during rotating shiftwork, and insufficient sleep are each independently associated with poor health outcomes, including obesity and glucose intolerance. A potential mechanism for poor health is increased energy intake (i.e., eating), particularly during the circadian night, when the physiological response to energy intake is altered. However, the contributions of CCD and insufficient sleep to subjective hunger, appetite, food preference, and appetitive hormones are not clear. To disentangle the influences of these factors, we studied seventeen healthy young adults in a 32-day in-laboratory study designed to distribute sleep, wakefulness, and energy intake equally across all phases of the circadian cycle, thereby imposing CCD. Participants were randomized to the Control (1:2 sleep:wake ratio, *n* = 8) or chronic sleep restriction (CSR, 1:3.3 sleep:wake ratio, *n* = 9) conditions. Throughout each waking episode the participants completed visual analog scales pertaining to hunger, appetite, and food preference. A fasting blood sample was collected to assess appetitive hormones. CCD was associated with a significant decrease in hunger and appetite in a multitude of domains in both the Control and CSR groups. This change in hunger was significantly correlated with changes in the ghrelin/leptin ratio. These findings further our understanding of the contributions of CCD and insufficient sleep on subjective hunger and appetite as well as of their possible contributions to adverse health behaviors.

## 1. Introduction

In the modern 24-h society, many work operations must be performed during times at which the internal circadian timing system is promoting inactivity, sleep, and fasting. Shiftwork, specifically with rotating work start and end times, is associated with increased risks for obesity [1,2], cardiovascular disease [3,4,5], glucose intolerance [6], and other adverse health outcomes. As shiftwork often requires an individual to initiate sleep during times when the internal biological clock is promoting wakefulness, disturbed and/or insufficient sleep [7], which can impair health [8], often occurs.

One proposed mechanism for adverse health outcomes during circadian disruption and insufficient sleep is energy intake at inappropriate circadian times. In-laboratory studies have demonstrated that when individuals are provided meals during the circadian night (when melatonin concentrations are elevated), they have a lower energetic response to that meal compared to eating during the day [9,10,11] as well as impaired glucose tolerance [11,12,13,14]. Individuals living at home who have energy intake later in the day have higher body compositions [15,16,17], lose less weight during weight-loss programs [18], and have increased cardiometabolic risk [19]. Many individuals living at home do not obtain sufficient sleep [20]. When volunteers are experimentally sleep-restricted with access to food *ad libitum*, they consume a greater number of calories than when not sleep restricted; these increases in calories typically occur in post-dinner snacks [21,22,23]. There is strong evidence, therefore, to suggest that energy intake later in the day may be contributing to poor health during both circadian disruption and insufficient sleep.

In-laboratory studies have demonstrated that subjective hunger and appetite exhibit a circadian rhythm, peaking during the circadian evening [24,25] at the same time as when sleep-restricted individuals consume a greater number of calories [21,22,23]. During conditions with sleep episodes lasting less than 6 h per night (i.e., chronic sleep restriction [CSR]) and in which participants are provided a diet designed to meet caloric needs; however, this rhythm is not altered [25]. This suggests that circadian timing may play a larger role in hunger rhythms than sleep restriction. It is unknown how multiple days with energy intake during the circadian evening and night and at a time when melatonin concentrations are elevated and promoting sleep (i.e., chronic circadian disruption [CCD]) may alter this hunger rhythm, appetite, and associated dietary preferences. Uncovering the influence of CRD in combination with CSR on hunger and dietary preferences is important, as there are conflicting reports regarding energy intake and food preference in shift workers and this is a pattern that is commonly observed in overnight or rotating shiftwork [26]. Hulsegge et al. have reported that shift workers have higher energy intake than day workers with similar diet quality [27], and Atkinson et al. have reported that shift work increases intake of calorie-dense, high-fat foods [28]. However, others have found through observational studies that daily energy intake in shift workers does not differ from dayworkers [29,30,31,32] or even that it is reduced [33]. Thus, rigorously identifying the contributions of CCD and CSR in tightly-controlled in-laboratory conditions, as compared to observational investigations, may provide insight into fundamental biological mechanisms that could be potentially targeted to improve health in shiftwork populations.

To uncover the impact of CCD, CSR, and their combination on hunger, dietary preferences, and physiological appetitive hormones, we studied healthy individuals who were randomized into either CSR or Control conditions within a ‘forced-desynchrony’ (FD) protocol with 20-h cycle lengths. Use of this protocol (20-h FD [34]) allowed for the endogenous circadian pacemaker to cycle at each participant’s endogenous ~24-h circadian period, thereby uncoupling sleep/wake and associated behaviors (occurring in 20-h cycles) from circadian (~24-h) influences, both individually and in combination, on subjective hunger and appetite as well as associated physiology.

## 2. Materials and Methods

Data were collected as part of a 32-day in-laboratory study to examine the relationships between CSR, circadian timing, and cognitive performance; primary outcomes have been previously published [34,35]. Written informed consent was obtained from each participant, and the Brigham and Women’s Hospital Partners Healthcare Institutional Review Boards approved all aspects of the protocol. The study was registered on clinicaltrials.gov as NCT01581125.

### 2.1. Participants

Inclusion criteria consisted of a self-reported habitual sleep duration between 7 h and 9 h (averaged across weekday/work days and weekend/free days), no night-shift work or travel across > 2 time zones < 3 months prior to study, a BMI between 18 and 29.9 kg/m^2^, aged between 18 and 35 years, not pregnant, and not using any prescription medications. To determine if volunteers met these inclusion criteria, participants underwent intensive medical and psychological screening including self-reported health and psychological screening questionnaires, physical examination by a physician, laboratory testing of hematological or metabolic measures, and a psychological evaluation from a clinical interview with a psychologist. Additionally, participants were determined to be free of any sleep disorders via questionnaires and an overnight clinical sleep screening.

### 2.2. Ambulatory Protocol

Prior to the in-laboratory protocol, participants maintained a ~10-h nightly sleep schedule at their self-reported habitual timing for at least three weeks. The duration and timing of this 10-h sleep schedule was verified by sleep logs, wrist actigraphy (Actiwatch-L Mini Mitter/Respironics), and call-ins to a time-stamped voicemail recording system immediately upon awakening and prior to going to bed. Throughout these ~three weeks and in-laboratory protocol, participants abstained from use of drugs, over-the-counter medication, alcohol, caffeine, nicotine, and other foreign substances, which was verified via urine toxicology upon in-laboratory admission.

### 2.3. In-Laboratory Protocol

Immediately following the ~three-week ambulatory protocol, participants were admitted to the Brigham and Women’s Hospital Center for Clinical Investigation to an environment free of time-cues and which was in dim lighting (<4 lux) during scheduled wakefulness and no lighting (0 lux) during scheduled sleep opportunities. All events were scheduled relative to the individual participant’s habitual sleep–wake timing as determined from the ambulatory monitoring.

To ensure participants had no residual insufficient sleep, the first three days of the protocol consisted of 12-h nighttime sleep opportunities and 4-h daytime sleep opportunities followed by two days consisting of 10-h sleep opportunities at habitual times. Participants were then scheduled to 24 cycles (i.e., “days”; these occurred over 20 calendar days) of a 20-h FD protocol and randomized to one of two sleep:wake FD conditions: Control (1:2, 6.67 h sleep opportunity, 13.33 h wake, equivalent to 8 h of sleep per 24 h day; 8 participants) or CSR (1:3.3, 4.67 h sleep, 15.33 h wake, equivalent to 5.6 h of sleep per 24 h day; 9 participants) (Supplemental Figure S1). Each 20-h “day” had identical patterns of meal timing, blood sample collection, questionnaire timing, and sleep/wake timing. The participants were blinded to specifics of the protocol (e.g., clock time, duration of wakefulness, day of study) and to which condition they were randomized (Control vs. CSR). During the 24 “days” of 20-h FD, individuals completed four ‘beat cycles’, defined as the time it took to complete the full range of timing relationships between sleep onset (20-h cycle) and circadian phase (~24.2-h cycle on average [36]). Thus, for the combination of 20-h and ~24.2-h, each beat cycle contained ~six protocol days (Supplemental Figure S1). Last, participants were scheduled five 24-h days of recovery (10 h sleep opportunities) at a similar circadian phase relationship as the beginning of the protocol. Wakefulness was established by continuous monitoring by study staff and polysomnographic recordings; participants were allowed to engage in sedentary activities during scheduled wakefulness (e.g., reading, conversing with research staff, watching movies).

Throughout the protocol, participants were provided with a nutritionist-designed isocaloric diet consisting of 45–50% carbohydrate, 30–35% fat, and 15–20% protein adjusted for sex, weight, and age using the Harris–Benedict equation with an activity factor of 1.3 [37]. Meals were provided at 01:25 h, 05:30 h, and 09:30 h post-awakening. Subjective hunger, appetite, and preference for certain foods were measured via visual analog scales that were provided before and after each meal and prior to each sleep opportunity. Specifically, the visual analog scales prompted participants to denote on a 100 mm horizontal line how they felt at that moment, with each end of the line labeled with “not at all” to “extremely”. Variables included asking about subjective hunger metrics (i.e., how hungry and thirsty a person thought they were, how much a person thought they could eat, what desire a person had to eat, and how full and how nauseous a person felt) as well as their subjective food preference (i.e., desire to eat sweet foods, salty foods, starchy foods, meats and poultry, fruits, and vegetables). To measure physiological changes in the appetitive hormones leptin and ghrelin, fasted blood was drawn via a venous catheter ~5 min after scheduled awakening. Core body temperature was continuously measured via rectal thermistor.

### 2.4. Assay Information

Assays for leptin and ghrelin were performed by the Brigham and Women’s Hospital Research Assay Core; the Core was blinded to study condition. Leptin and active ghrelin were assayed using serum radioimmunoassay techniques (Millipore Research, St. Charles, MO, USA). The leptin assay had a sensitivity of 0.1 ng/mL, a within-assay coefficient of variation (CV) of 5.2–5.7% and a between assay CV of 3.2–8.9%; ghrelin had a sensitivity of 7.8 pg/mL, a within CV of 6.5–9.5% and a between CV of 9.6–16.2%.

### 2.5. Statistical Analysis

Data were binned and analyzed as average values per participant within each beat cycle (range of 36–48 visual analog records per beat cycle), circadian phase, and time awake (i.e., response to study meals). Circadian phase was determined via non-orthogonal spectral analysis [38] of core body temperature to estimate intrinsic circadian period and subsequent circadian phase (0^0^ = core body temperature minimum). To test for the impact of CCD with or without CSR, a linear mixed model analysis was used with the beat cycle, condition, and their interaction as fixed factors and the participant as a random factor. Mixed model analyses were used to detect group differences by circadian phase or time into wakefulness. Paired *t*-tests were used to compare subjective appetite, food preference, and appetitive hormones in Beat cycle 1 vs. Beat cycle 4 to uncover the chronic aspect of CCD. To correct for multiple *t*-test planned comparisons, a modified Bonferroni correction was applied to reduce the type one error (*p* < 0.033 needed for significance) [39]. To examine how a change in appetitive hormones across CCD and CSR might be influencing subjective hunger, Pearson correlations were used to test for relationships between changes in ghrelin/leptin ratio [40] and subjective hunger between Beat cycles 1 and 4. Fasted blood samples were unable to be obtained from one participant in the CSR condition, and the change score in ghrelin/leptin ratio was deemed to be an outlier (interquartile range × 1.5) in three participants (one Control and two CSR participants); thus, thirteen participants were analyzed in the Pearson correlation. All statistical analyses were performed using SAS 9.4.

## 3. Results

Seventeen healthy participants (seven male; aged 26.1 ± 4.4 y, 20.0 to 34.0 y; body mass index (BMI), 24.0 ± 3.6 kg/m^2^, 18.2 to 28.4 kg/m^2^ [mean ± SD, range]) completed the in-laboratory protocol (Supplemental Table S1). Of the seventeen participants, eight participants had a BMI classified as overweight (i.e., 25–30 kg/m^2^), with three of those participants randomized to the Control condition and five to the CSR condition. There were no significant differences in BMI between the Control and CSR groups (Supplemental Table S1).

### 3.1. Impact of Chronic Sleep Restriction and Circadian Disruption on Subjective Hunger, Thirst, Satiety, and Nausea

**Figure 1:**
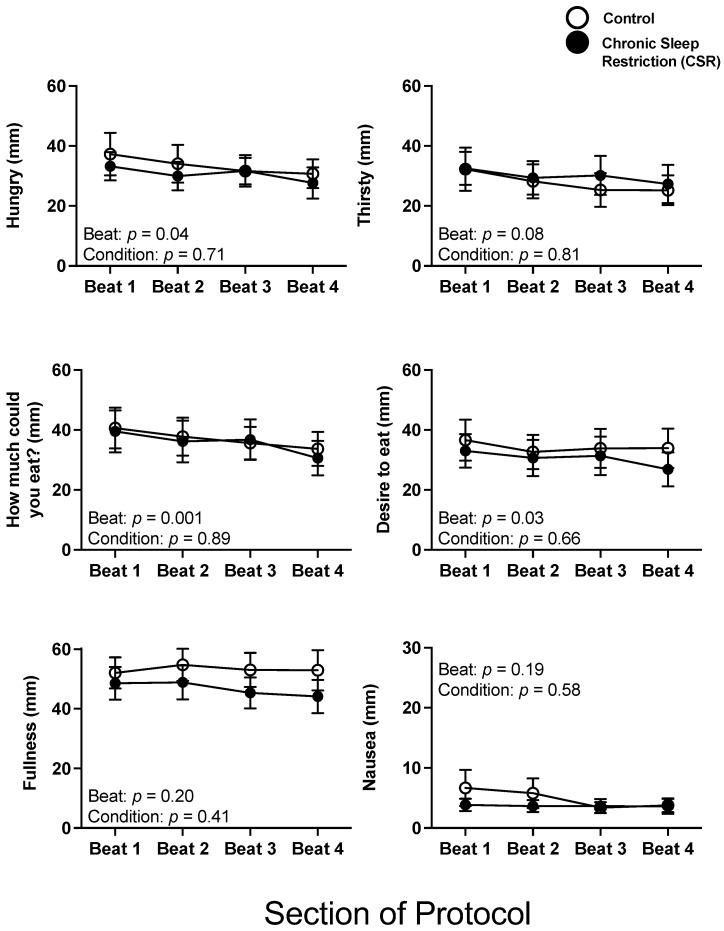
Acute and chronic impact of circadian disruption and sleep restriction on subjective hunger and appetite. Data from the Control (*n* = 8, 1:2 sleep:wake ratio) participants are denoted by open circles and those from the Chronic Sleep Restriction (CSR, *n* = 9, 1:3.3 sleep:wake ratio) participants are denoted by closed circles. Higher scores indicate higher subjective feelings of each aspect of hunger and appetite; the possible range was 0–100. Beat cycles (i.e., time to complete a cycle of circadian and sleep:wake schedule combinations, which was ~6 protocol days in this forced desynchrony design) of the protocol are shown on the *x*-axis. Error bars represent SEM. Note, the *y*-axis scale of Nausea is one-half the range of the others. *p* values are derived from linear mixed models with beat cycle, condition, and their interactions as fixed effects and participant as a random factor.

There were no significant interaction or condition effects between the Control and CSR conditions across beat cycles for subjective hunger, thirst, satiety, and nausea (all *p* > 0.25). There were significant beat cycle effects for hunger and several subjective satiety outcomes, such that hunger and satiety decreased across beat cycles (Figure 1).

**Figure 2:**
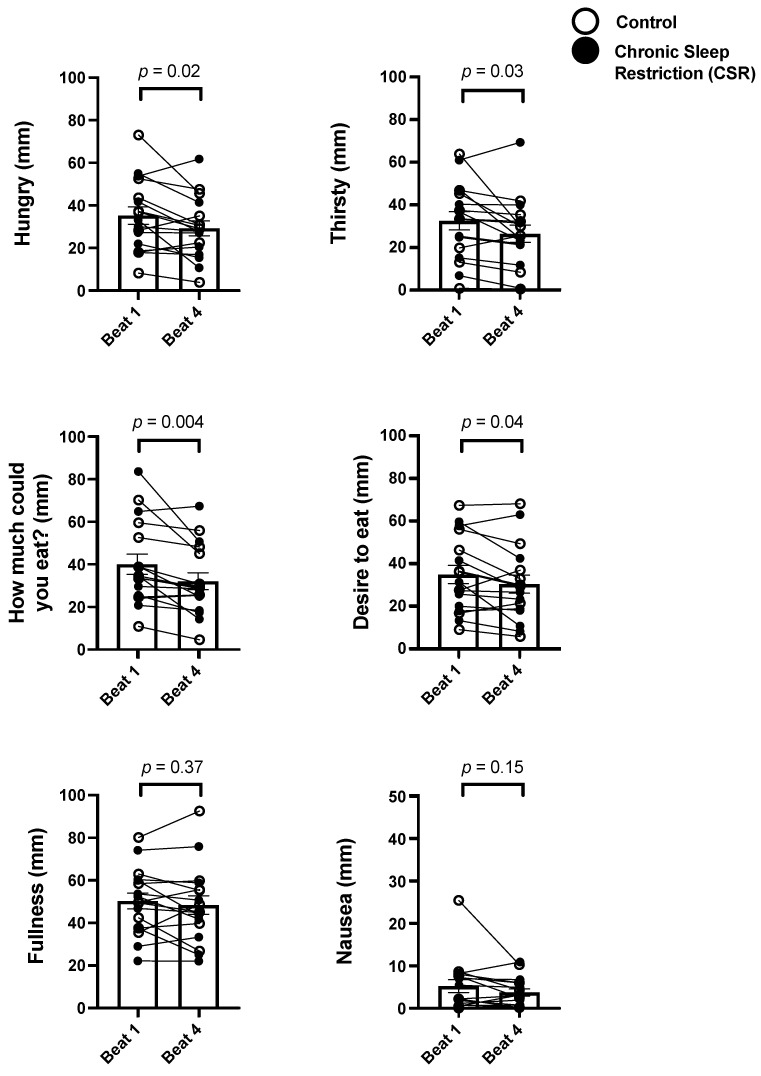
Chronic impact of circadian disruption on subjective hunger and appetite. Data from the Control (*n* = 8, 1:2 sleep:wake ratio) participants are denoted by open circles and those from the Chronic Sleep Restriction (CSR, *n* = 9, 1:3.3 sleep:wake ratio) participants are denoted by closed circles. Higher scores indicate higher subjective feelings of each aspect of hunger and appetite. Beat cycles (i.e., time to complete a cycle of circadian and sleep:wake schedule combinations, which was ~6 protocol days in this forced desynchrony design) of the protocol are shown on the *x*-axis. Group means are each beat cycle are denoted by the open column and error bars represent SEM. Note, the *y*-axis of Nausea is approximately one-half the range of the others. *p* values are derived from independent *t*-tests used to test differences between Beat cycle 1 and Beat cycle 4.

As there were no condition effects, we combined the groups and analyzed change in subjective scores from Beat cycle 1 to 4 to test the effects of CCD. There was a significant decrease in subjective hunger (−17.1%; *p* = 0.02), thirst (−18.8%; *p* = 0.03), and how much a person thought they could eat (−19.9%; *p* = 0.004) (Figure 2) as well as a non-significant trend for a decrease in the strength of desire to eat (−13.0%; *p* = 0.04). There were no significant changes in the participants subjective fullness (*p* = 0.37) or nausea (*p* = 0.15) across CCD (Figure 2).

There were no significant interaction or condition effects between the Control and CSR groups in subjective thirst, satiety, and nausea across circadian phase or time into wakefulness across the protocol (all *p* > 0.11; Supplemental Figure S2). How much a person thought they could eat, the strength of desire to eat, and nausea all had significant circadian phase effects; there was no circadian effect for thirst (Supplemental Figure S2). All hunger and satiety outcomes differed by time into wakefulness, with changes occurring between meals (Supplemental Figure S2).

### 3.2. Impact of Chronic Sleep Restriction and Circadian Disruption on Food Preference

**Figure 3:**
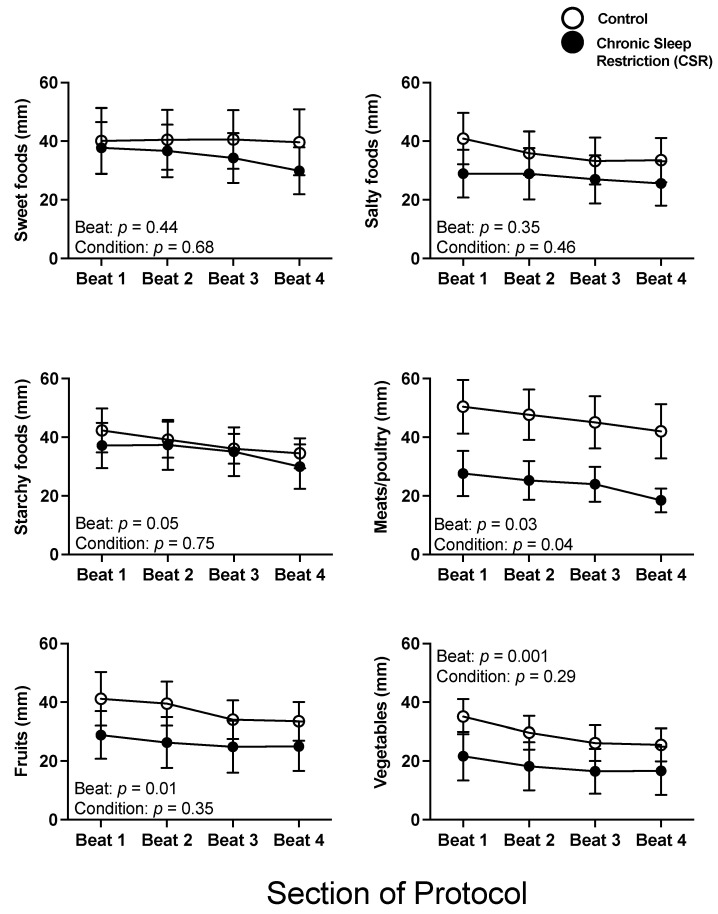
Acute and chronic impact of circadian disruption and sleep restriction on subjective food preference. Data from the Control (*n* = 8, 1:2 sleep:wake ratio) participants are denoted by open circles and those from the Chronic Sleep Restriction (CSR, *n* = 9, 1:3.3 sleep:wake ratio) participants are denoted by closed circles. Higher scores indicate higher subjective feelings of each aspect of food preference; the possible range was 0–100. Beat cycles (i.e., time to complete a cycle of circadian and sleep:wake schedule combinations, which was ~6 protocol days in this forced desynchrony design) of the protocol are shown on the *x*-axis. Error bars represent SEM. *p* values are derived from linear mixed models with beat cycle, condition, and their interactions as fixed effects and participant as a random factor.

There were no significant interaction or condition effects between the Control and CSR conditions across beat cycles for subjective food preference (all *p* > 0.29), with the exception of the Control condition having a significantly higher preference for meats/poultry across beat cycles compared to the CSR condition (*p* = 0.04). There were significant beat cycle effects on preference for meats/poultry, fruits, and vegetables, and a non-significant trend in preference for starchy foods (*p* = 0.05), such that these preferences decreased across beat cycles (Figure 3).

**Figure 4:**
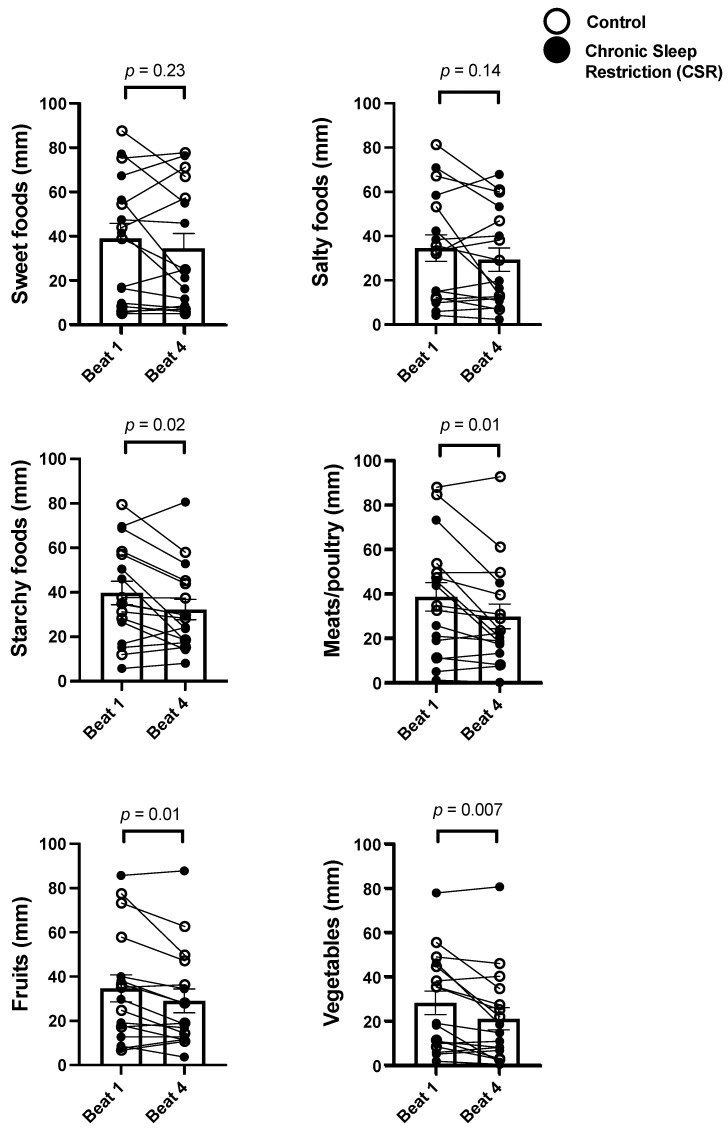
Acute and chronic impact of circadian disruption on food type preference. Data from the Control (*n* = 8, 1:2 sleep:wake ratio) participants are denoted by open circles and those from the Chronic Sleep Restriction (CSR, *n* = 9, 1:3.3 sleep:wake ratio) participants are denoted by closed circles. Higher scores indicate higher subjective feelings of each aspect of food preference. Beat cycles (i.e., time to complete a cycle of circadian and sleep:wake schedule combinations, which was ~6 protocol days in this forced desynchrony design) of the protocol are shown on the *x*-axis. Group means of each beat cycle are denoted by the open column and error bars represent SEM. *p* values are derived from independent *t*-tests used to test differences between Beat cycle 1 and Beat cycle 4.

The Control and CSR groups were then combined and analyzed across beat cycles to determine the influence of CCD. While there were no significant changes in the participants’ subjective preference for sweet (*p* = 0.23) or salty (*p* = 0.14) foods across CCD, there were significant decreases in subjective preference for starchy foods (−18.9%; *p* = 0.02), meats/poultry (−23.0%; *p* = 0.01), fruits (−16.2%; *p* = 0.01), and vegetables (−25.7%; *p* = 0.01) (Figure 4).

There was a significant circadian phase by condition interaction for preference for starchy foods, a non-significant trend for circadian phase by condition interaction for preference for sweet foods (*p* = 0.06), and a significant condition effect across both circadian phase and time awake for higher meats/poultry preference in the Control group (Supplemental Figure S3). All food preference outcomes had significant circadian phase effects and time into wakefulness effects (Supplemental Figure S3).

### 3.3. Relationship between the Change in Hunger and Ghrelin/Leptin Ratio

**Figure 5:**
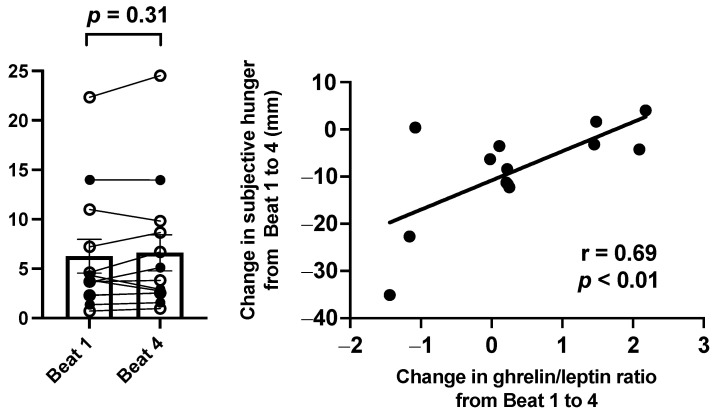
Impact of circadian disruption on ghrelin/leptin ratio and the relationship between the change in subjective hunger on a visual analog scale and the change in fasted ghrelin/leptin ratio across chronic circadian disruption (Beat cycle 1 to Beat cycle 4). Data from the Control (*n* = 8, 1:2 sleep:wake ratio) participants are denoted by open circles and those from the Chronic Sleep Restriction (CSR, *n* = 9, 1:3.3 sleep:wake ratio) participants are denoted by closed circles in the (**right**) panel. Beat cycles (i.e., time to complete a cycle of circadian and sleep:wake schedule combinations, which was ~6 protocol days in this forced desynchrony design) of the protocol are shown on the *x*-axis. Group means for each beat cycle are denoted by the open column and error bars represent SEM. *p* values are derived from independent *t*-tests used to test differences between Beat cycle 1 and Beat cycle 4. In the (**left**) panel, data points represent individual participants and the solid line represents the linear fit of the data. *p* values derived from Pearson correlation.

There were no differences between the Control and CSR groups in ghrelin/leptin ratio during Beat cycle 1 (*p* = 0.49) or Beat cycle 4 (*p* = 0.38). Additionally, there were no significant differences in change in ghrelin/leptin ratio across beat cycles between the groups (*p* = 0.21) or within the entire sample (*p* = 0.31; Figure 5).

To explore the relationship between the appetitive hormones and subjective hunger across CCD, the change in ghrelin/leptin ratio was correlated with the change in subjective hunger scores. There was a significant positive association between change in ghrelin/leptin ratio and change in subjective hunger (r^2^ = 0.48; *p* < 0.01; Figure 5) such that an increase in the ratio was associated with less of a decrease in subjective hunger.

## 4. Discussion

Identifying mechanisms by which insufficient sleep and circadian disruption influence behaviors that may promote poor health is necessary for designing effective treatment strategies. In our tightly-controlled randomized single-blind in-laboratory study that induced both CSR and CCD, we discovered that while CSR has minimal effect on subjective hunger, appetite, and food preference, CCD typically decreases these subjective outcomes. Moreover, while we did not find any influence in CSR or CCD on the fasting levels of physiological appetitive hormones, we did find that changes in these fasted appetitive hormones across CCD were tightly correlated with changes in subjective hunger ratings. Taken together, these findings provide insight into the interplay between CSR and CCD in controlled diet settings.

Our findings of no difference in subjective hunger or appetite ratings (i.e., desire to eat, fullness) between the Control and CSR groups across beat cycles was to a certain extent in line with our previous report from the current study population of no difference in hunger between groups across circadian phase [25], although at odds with other reports of increases in these outcomes under acute (1–2 nights) sleep restriction [40,41,42]. Interestingly, when participants are provided access to food *ad libitum* during controlled acute sleep restriction (five days), hunger and appetite levels decrease, yet actual food consumption increases [21], suggesting other physiological or psychological changes are likely responsible for the observations of increased energy intake during sleep restriction [21,43]. Furthermore, we are now able to add to the literature on the impact of CCD on subjective hunger and appetite; namely, a significant decrease in these outcomes across our 20-day FD protocol. One potential explanation for these decreases is that participants may have been in a state of positive energy balance. Energy expenditure has been reported to be decreased during circadian misalignment [9] and CSR coupled with CCD [11], as in the current protocol. Thus, as the caloric content of the in-laboratory meals in this current study were tightly-controlled to meet participants’ specific energetic needs, if energy expenditure were to have decreased, as previously reported in [9,11], the current study participants would have been in a positive energy balance and thereby exhibited feeling of reduced hunger and appetite. Future work is needed to elucidate the relationship between eating habits and hunger levels in chronic shift worker populations when energy intake is not controlled in order to determine whether this relationship persists in real-world settings.

In addition to subjective hunger and appetite, we examined how CSR and CCD influenced subjective preference for certain types of foods. It has been well-established that individuals that are sleep restricted tend to choose foods that are of poorer quality compared to non-sleep restricted individuals [22,23,44,45]. Additionally, when the brain is imaged utilizing fMRI techniques, sleep-restricted individuals’ brains show higher activation to unhealthier food options that are higher in caloric content [46,47]. In regards to circadian disruption, and as mentioned above, there have been conflicting reports regarding diet quality [26]. Thus, understanding food preference during controlled sleep and CCD may provide further insight into how changes to these processes could influence diet quality independent of exogenous factors in observational studies (e.g., food availability [48]). Our findings of no differences in food preferences between sleep groups with the exception of meats/poultry and of decreases in preference for most food types across CCD may be a byproduct of the overall reduction in hunger and appetite.

Though we did not find differences in fasted ghrelin/leptin ratio between the control and CSR groups or across CCD, we did find a significant association between change in ghrelin/leptin ratio and the change in subjective hunger scores across beat cycles. Thus, though subjective hunger decreased across CCD (potentially motivated by the aforementioned theoretical change in energy balance), this decrease may have been driven by physiological changes to the appetitive hormones. This suggests that the decrease in subjective hunger was likely not solely due to the limited (and repeated) dietary options during the protocol. Similar findings of a change in ghrelin/leptin ratio and hunger have been reported across a baseline and sleep restriction [40], and the change in ghrelin during sleep restriction has been found to be correlated with energy intake, particularly from sweet foods (i.e., candy) [49]. We have previously reported that fasted leptin and ghrelin are not independently different during CSR across circadian phases [25], and others have shown leptin to be reduced [9,50,51] and ghrelin increased during acute circadian misalignment [52], though these findings may be sex-dependent [53]. Thus, physiological changes in appetitive hormones may be a mechanism for changes in behavior (i.e., hunger and eating) during CCD.

Despite our rigorous randomized design in tightly-controlled laboratory settings, there are several limitations to consider. As stated previously with observations of eating behaviors during sleep restriction, subjective hunger and appetite feelings do not always match objective eating patterns [21,22,23]. Thus, it may be difficult to extrapolate our findings into real-world outcomes. However, understanding physiology in a controlled manner such as the current protocol is necessary for identifying fundamental relationships. Second, the current protocol only consisted of collection of fasted hormones upon awakening, with no measurement of appetitive hormones in the postprandial state. This may be of importance, as hunger during sleep restriction differs depending on the pre- or postprandial state [41], which could coincide with changes in these hormones. Third, other exogenous factors beyond CCD were not measured and could have contributed to the reductions in hunger and appetite, such as the length of the protocol and repeated hospital meals. Future work in habitual settings could help account for this limitation. Fourth, several of our participants met the criteria for an overweight body composition, which has been shown to alter levels of leptin and ghrelin during both fasted [54,55] and postprandial [54] states. There were no significant differences in body composition between Control and CSR groups. In addition, the within-design analysis across CCD that we used controlled for a body composition effect. Including participants with an overweight body composition may improve the generalizability of our findings. Last, no current night or rotating shift workers were included; future work in these and other similar populations should be conducted.

## 5. Conclusions

In summary, our findings further our understanding of the contributions of CCD and CSR and of the hormones leptin and ghrelin on subjective and physiological drivers of eating behavior. Importantly, our findings of decreases in most measured outcomes of subjective hunger, appetite, and food preference in our highly controlled conditions likely suggest that other mechanisms promote the observed adverse health consequences of shift work.

## Data Availability

The raw data supporting the conclusions of this manuscript will be made available by the authors, upon request, to any qualified researcher.

## Supplementary Materials

The following supporting information can be downloaded at: https://www.mdpi.com/article/10.3390/nu14091800/s1, Figure S1: Example of the study protocols for the Control (*n* = 7, 1:2 sleep:wake ratio, equivalent to 8 h sleep opportunity per 24 h day) and Chronic Sleep Restriction (*n* = 8, 1:3.3 sleep:wake ratio, equivalent to 5.6 per 24 h day) condition across recurrent circadian disruption; Figure S2. Impact of condition, circadian phase, and time awake on subjective hunger and satiety measures.; Figure S3. Impact of condition, circadian phase, and time awake on subjective food preference, Table S1. Participant demographics.
