# Supplementary material for: Chronic Circadian Disruption and Sleep Restriction Influence Subjective Hunger, Appetite, and Food Preference
Source: Nutrients. 2022 Apr 26;14(9):1800. doi: 10.3390/nu14091800 (PMC9105437; doi:10.3390/nu14091800)
Supplement: Supplementary file 1 [file nutrients-14-01800-s001.zip › nutrients-1639145-supplementary.pdf]

nutrients

MDPI

Supplemental Information

Chronic Circadian Disruption and Chronic Sleep Restriction Influence Subjective Hunger and Appetite

Andrew W McHill[1,2,3,4*], Joseph T Hull[3,4] and Elizabeth B Klerman[3,4,5]

[1] Sleep, Chronobiology, and Health Laboratory, School of Nursing, Oregon Health & Science University, Portland OR, USA.
[2] Oregon Institute of Occupational Health Sciences, Oregon Health & Science University, Portland OR, USA.
[3] Division of Sleep Medicine, Harvard Medical School, Boston, MA, USA.
[4] Division of Sleep and Circadian Disorders, Departments of Medicine and Neurology, Brigham and Women's Hospital, Boston, MA, USA.
[5] Department of Neurology, Massachusetts General Hospital, Boston, MA, USA.
* Correspondence: Andrew W. McHill, Sleep, Chronobiology, and Health Laboratory, School of Nursing, Oregon Health & Science University, 3455 SW US Veterans Hospital Rd, Portland, OR 97239, USA. Email: mchill@ohsu.edu.

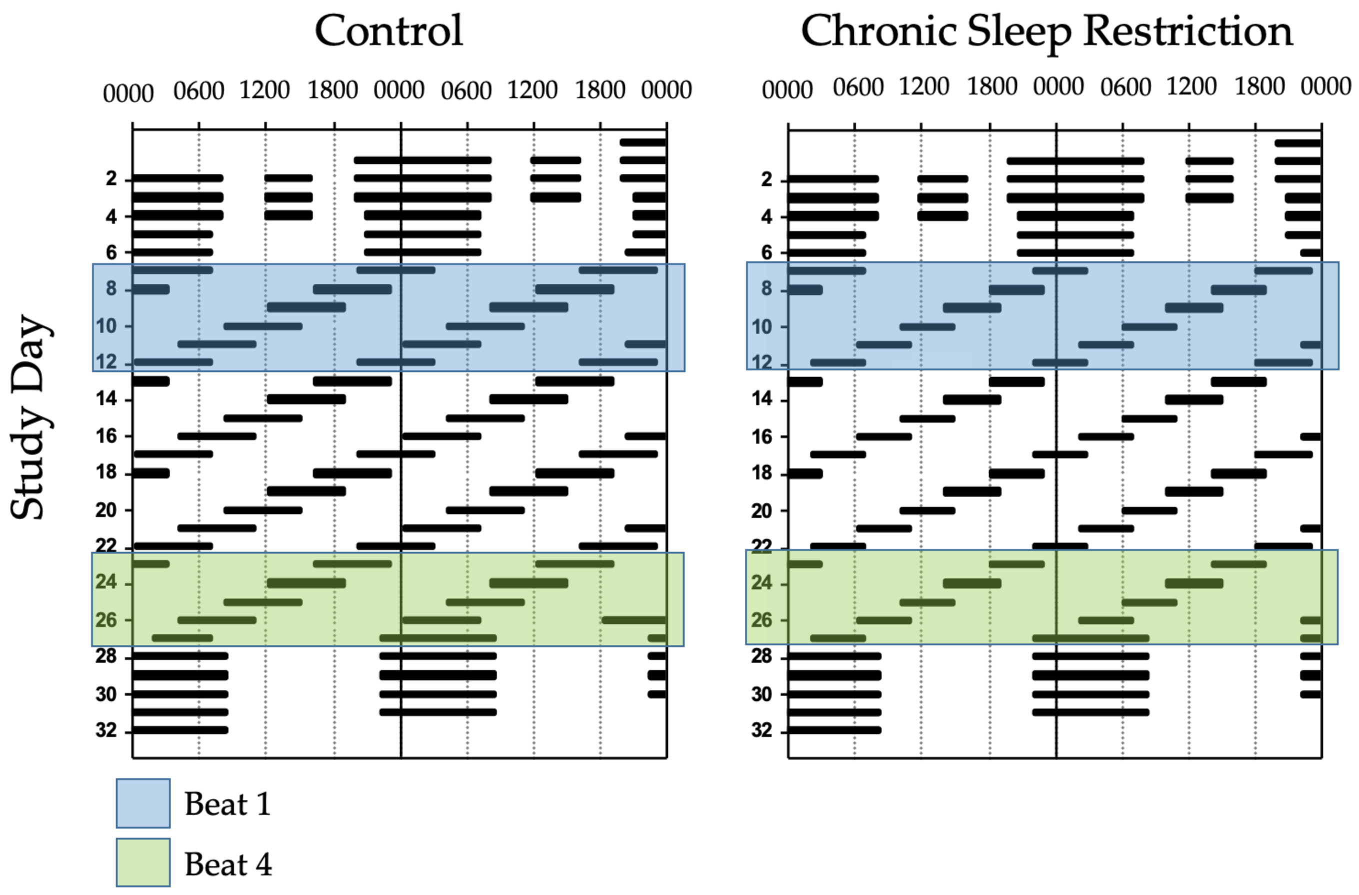

Supplemental Figure S1. Example study protocols for the Control (n = 7, 1:2 sleep:wake ratio, equivalent to 8 h sleep opportunity per 24 h day) and Chronic Sleep Restriction (n = 8, 1:3.3 sleep:wake ratio, equivalent to 5.6 per 24 h day) conditions across recurrent circadian disruption. Days are double plotted; g each study day is plotted next to and below the previous day. Clock time is plotted on the top x-axis and study day down the y-axis. Solid black bars represent sleep opportunities and the shaded blue and green rectangles indicate Beat cycles 1 and 4, respectively (approximately 6 protocol days). Exact timing of all events for each participant depended on their habitual sleep and wake times immediately before the inpatient protocol.

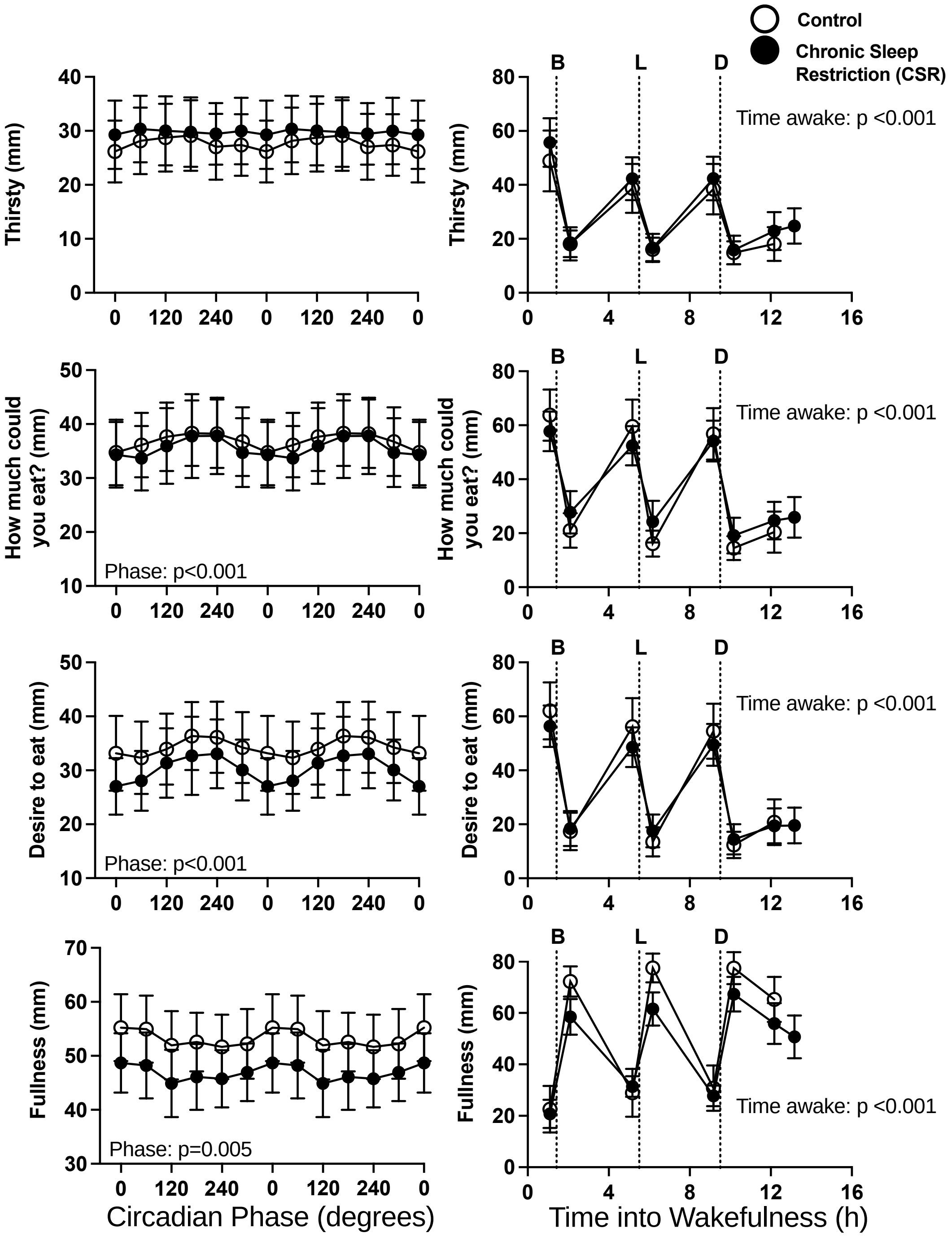

Supplemental Figure S2. Impact of condition, circadian phase, and time awake on subjective hunger and satiety measures. Data from the Control (n = 8, 1:2 sleep:wake ratio) participants are denoted by open circles and from the Chronic Sleep Restriction (CSR, n = 9, 1:3.3 sleep:wake ratio) participants are denoted by closed circles. Higher scores indicate higher subjective feelings of hunger and satiety. Circadian phase is derived from core body temperature minimum (0 degrees). Dashed lines in the right column represent meal times, with B indicating breakfast, L indicating lunch, and D indicating dinner. Error bars represent SEM. P values are derived from linear mixed models with circadian phase or time awake, condition, and their interactions as fixed effects and participant as a random factor. Note, the y-axis in the right column is twice the range of the left column.

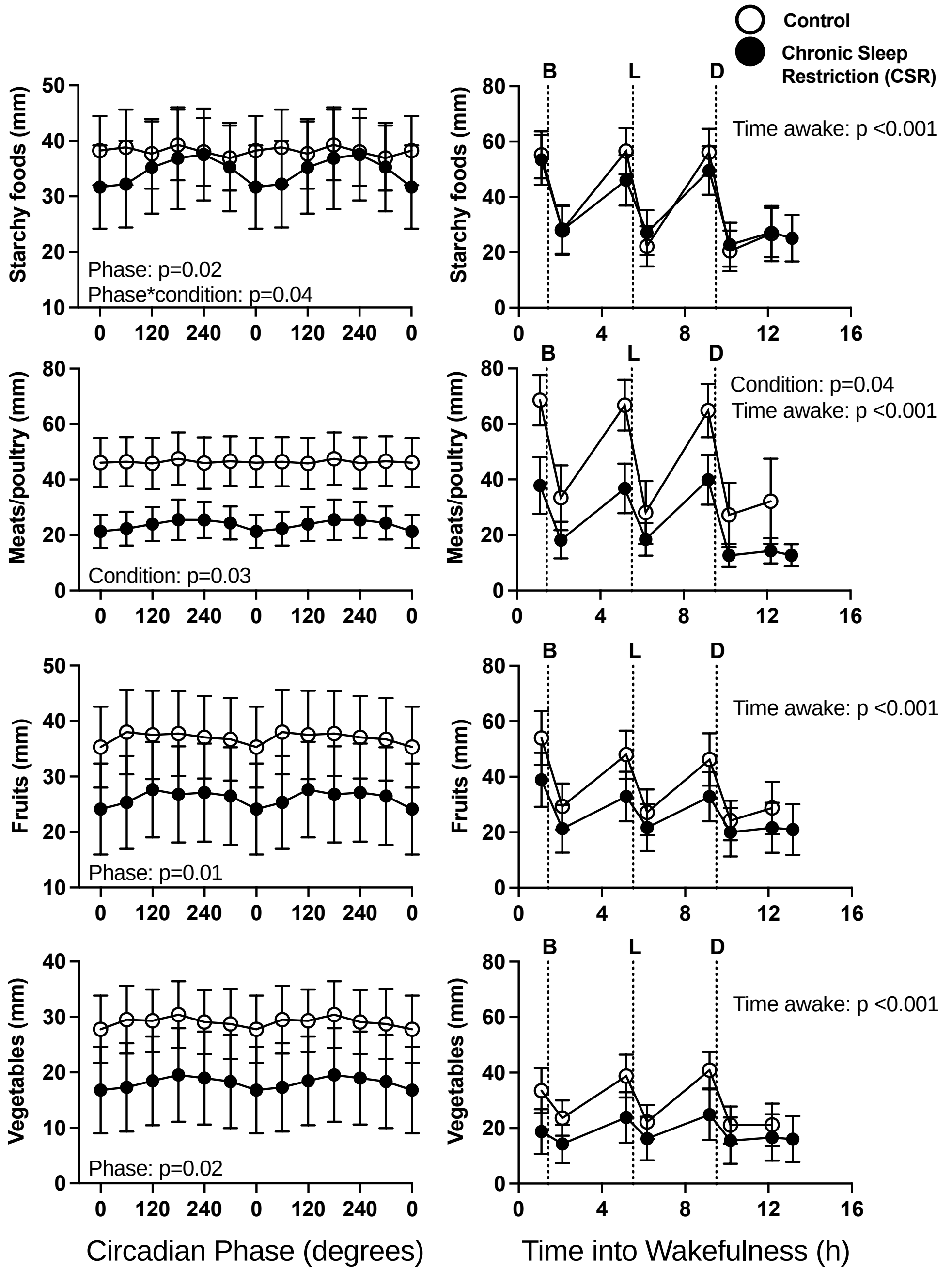

Supplemental Figure S3. Impact of condition, circadian phase, and time awake on subjective food preference. Data from the Control (n = 8, 1:2 sleep:wake ratio) participants are denoted by open circles and from the Chronic Sleep Restriction (CSR, n = 9, 1:3.3 sleep:wake ratio) participants are denoted by closed circles. Higher scores indicate higher subjective feelings of food preference. Circadian phase is derived from core body temperature minimum (0 degrees). Dashed lines in the right column represent meal times, with B indicating breakfast, L indicating lunch, and D indicating dinner. Error bars represent SEM. P values are derived from linear mixed models with circadian phase or time awake, condition, and their interactions as fixed effects and participant as a random factor. Note, the y-axis in the right column is twice the range of the left column.

Supplemental Table S1. Participant demographics.

	Control (n=8)	CSR (n=9)	p-value
**Sex and Age**			
Female, Male (#, %)	5, 3 (0.6, 0.4)	5, 4 (0.6, 0.4)	-
Age (years, range)	27.6 (22.0-34.0)	24.8 (20.0-30.0)	0.20
**Body Composition**			
Weight (kg, range)	64.3 (47.2-85.4)	68.2 (57.8-82.8)	0.52
Body Mass Index (kg/m^2, range)	23.1 (18.2-28.4)	24.8 (19.2-28.3)	0.36
Body Mass Index <25 kg/m^2 (#, %)	5 (0.6)	4 (0.4)	-
Body Mass Index >25 kg/m^2 (#, %)	3 (0.4)	5 (0.6)	-

p-values were derived via independent t-tests between groups.
